# Impact of peritumoral brain edema on pre- and postoperative clinical conditions and on long-term outcomes in patients with intracranial meningiomas

**DOI:** 10.1186/s40001-022-00962-y

**Published:** 2023-01-21

**Authors:** Hajrullah Ahmeti, Amke Caliebe, Christoph Röcken, Olav Jansen, Maximilian H. Mehdorn, Michael Synowitz

**Affiliations:** 1 Department of Neurosurgery, University Hospital Schleswig-Holstein, Campus Kiel, Arnold-Heller-Str. 3, 24105 Kiel, Germany; 2 Institute of Medical Informatics und Statistics, University Hospital Schleswig-Holstein, Kiel University, Kiel, Germany; 3 Department of Pathology, University Hospital Schleswig-Holstein, Campus Kiel, Kiel, Germany; 4 Department of Radiology and Neuroradiology, University Hospital Schleswig-Holstein, Campus Kiel, Kiel, Germany

**Keywords:** Brain edema, Intracranial meningiomas, Meningioma surgery, Neurological conditions, Long-term outcomes

## Abstract

**Background:**

Peritumoral brain edema (PTBE) is a common complication related to intracranial meningiomas. In several studies, researchers have investigated the pathogenesis of PTBE, and the factors involved in its development in patients with intracranial meningiomas have been reported. However, very little is known about the clinical effect of PTBE on patients with intracranial meningiomas; therefore, a systematic examination of this matter is necessary.

**Methods:**

In this study, we performed a systematic examination of 696 patients with primary intracranial meningiomas to assess the effect of preoperative PTBE on preoperative symptoms, neurological deficits and postoperative complications, and long-term outcomes with a follow-up period of 16.8 years. We performed a univariate analysis and multiple regression for specific outcomes and adjusted for other relevant clinical factors.

**Results:**

A total of 627 (90.1%) patients were symptomatic preoperatively. One hundred eighty-eight (90.8%) patients with small to moderate PTBE and 125 (98.4%) patients with severe PTBE presented with symptoms significantly more often than the 314 (86.7%) patients without PTBE (*p* < 0.001, univariate analysis). Cognitive deficits, palsy and seizure were significantly more present, preoperatively, in patients with PTBE than in patients without PTBE (*p* < 0.001, univariate analysis). Two hundred fifty-five (36.6%) patients experienced surgical and systemic complications postoperatively. The complication rate was significantly higher in patients with PTBE; 41.5% for patients with small to moderate PTBE and 52.8% for patients with severe PTBE, compared to 28.2% of patients without PTBE (*p* < 0.001, univariate analysis). Furthermore, pre- and postoperative KPS scores were significantly lower in patients with PTBE (*p* < 0.001). Patients with PTBE required additional medical support significantly more often (*p* < 0.001) and had a significantly longer hospital stay (*p* < 0.001). The mortality rate was higher in patients with PTBE immediately after surgery and in the follow-up period; however, the difference was not significant. The neurological condition of all patients improved in the follow-up and did not show significant differences between patients with and without preoperative PTBE (*p* = 0.6361). Multiple logistic regression analyses revealed a significant association between PTBE and the presence of preoperative cognitive deficits, the incidences of seizure and postoperative complications, and low pre- and postoperative KPS scores.

**Conclusions:**

Preoperative PTBE significantly increased the incidences of specific preoperative symptoms, neurological deficits and postoperative complications in patients with intracranial meningiomas. After surgery, patients with preoperative PTBE required medical support significantly more often than patients without PTBE. However, all patients had favorable outcomes after surgery.

**Supplementary Information:**

The online version contains supplementary material available at 10.1186/s40001-022-00962-y.

## Introduction

Meningiomas are, in most cases, benign, slowly growing tumors [1–3]. Due to this slow growth, some meningiomas can grow into a very large tumor mass before becoming symptomatic. In addition to their own tumor mass, meningiomas develop peritumoral brain edema (PTBE) in up to 78% of cases, which can occur in very different extensions and can lead to an additional increase in the space-occupying effect of meningiomas [4–6]. Regarding the pathogenesis of PTBE in intracranial meningiomas, many influencing factors, including patient age, sex, tumor site and size, irregular tumor margin, disruption of the arachnoid plane, pial blood supply, signal intensity on T2-weighted MRI, Ki-67 index, histological subtype, and tumor grade, have been reported, some with very contradictory results [5–12]. Furthermore, different theories on the development of PTBE in patients with intracranial meningiomas, such as compression of the brain parenchyma by large tumors causing ischemia and cytotoxic edema; compression of vessels, in particular large veins and sinuses, decelerating blood outflow; secretory theories describing the release of eosinophilic and periodic acid-Schiff positive inclusions and perivascular and angiogenic factors, such as vascular endothelial growth factor A (VEGF-A), resulting in increasing permeability of vessel walls; hydrodynamic theories based on hypoplastic tumor vessels; and hormonal theories involving meningiomas with progesterone receptor positivity, have been proposed [6, 13–16]. However, very few studies have reported the impact of PTBE on pre- and postoperative clinical conditions, neurological deficits and outcomes [17, 18]. There are some reports on the increased risk of pre- and postoperative seizures in meningioma patients with PTBE [19–21]. An increased risk of postoperative neurological deficits, increased mortality rates and poorer overall survival have also been reported [22–24]. However, these studies have very different conclusions. Furthermore, there are no publications in which researchers systematically examine the pre- and postoperative clinical effects of PTBE in patients with intracranial meningiomas. In addition, most reported studies on the clinical effect of PTBE in patients with intracranial meningiomas have a small number of cases, and in most series, there are also no volumetric measurements of preoperative PTBE, but mostly diametric data or only investigator-dependent assessments of PTBE, such as “not present”, “moderate” and “severe”.

To fill this gap in information, we performed a systematic investigation of the clinical impact of preoperative PTBE in a large cohort of patients with intracranial meningiomas. Outcome measures were pre- and postoperative symptoms, neurological conditions and long-term outcomes, taking into account all relevant other clinical factors, such as preexisting diseases and ASA score, as well as an accurate volumetric measurement of the preoperative PTBE in each patient. We finally show that preoperative PTBE significantly impacts the risk for specific preoperative symptoms, neurological deficits and postoperative complications in patients with intracranial meningiomas.

## Methods

From our archive, we retrieved all patients who had undergone surgery for primary intracranial meningioma between 2003 and 2019. Patients were included if intracranial meningioma was confirmed histologically according to WHO criteria [25, 26] and volume determination of PTBE was available. In patients without digital image files, the PTBE volume could not be determined. This patients were excluded from the study. The cohort was then divided into three groups: patients without PTBE, patients with an edema volume lower than the mean value of the edema volume (< 51.95 cm^3^, classified as small to moderate PTBE) and patients with an edema volume equal to or larger than the mean value of the edema volume (**≥ **51.95 cm^3^, classified as severe PTBE).

**Fig. 1:**
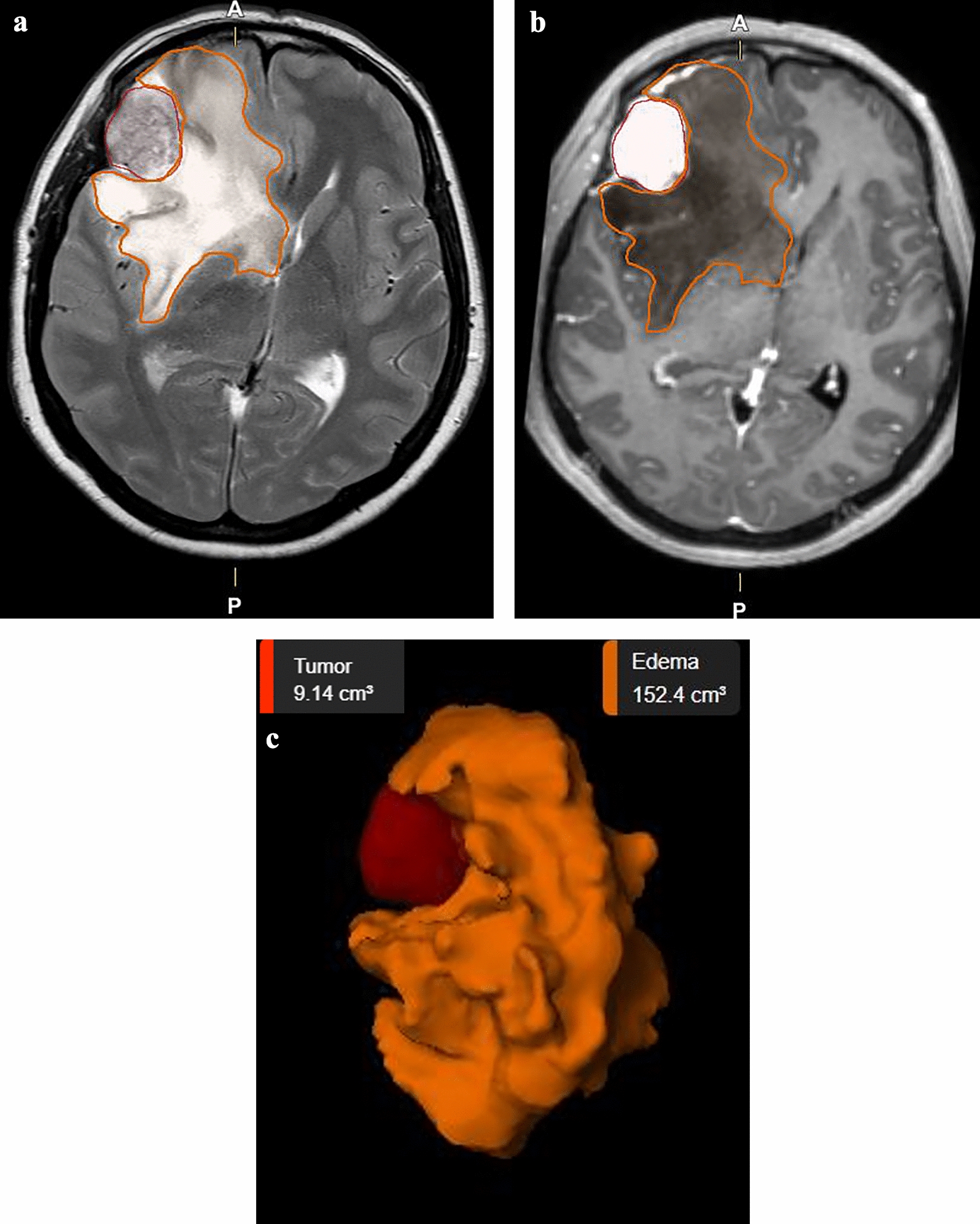
**A** Axial T2-weighted MRI and **B** T1-weighted MRI after gadolinium administration with right sphenoid wing meningioma (marked red) and extended PTBE (marked orange). **C** Schematic presentation of tumor volume and PTBE volume using the Brainlab^®^ neuronavigation system, Brainlab AG, Germany

All relevant clinical data were recorded, including age, sex, comorbidities, American Society of Anesthesiologists score (ASA score), tumor site, tumor volume, PTBE volume, pre- and postoperative symptoms and neurological conditions according to the Karnofsky performance status scale (KPS), extent of tumor resection according to Simpson grading, all postoperative complications within 30 days, histology and follow-up data. Tumor and PTBE volumes were accurately measured using the SmartBrush^®^ function (version 4.0) from the Brainlab^®^ neuronavigation system (Brainlab AG, Germany). Tumor volumes were determined on T1-weighted MRI after administration of gadolinium, and PTBE volumes were determined on T2-weighted MRI in cm^3^ (Fig. 1). Only for those patients without preoperative MRI, CT imaging were used to determine tumor and PTBE volumes. There were very few patients with only preoperative CT images. In addition, we analyzed the shape of the tumor, peritumoral rim, contrast enhancement behavior of the tumor, signal intensity of the tumor on T2 weighted MRI and blood supply of the tumor. All patients received dexamethasone perioperatively. Patients without or with small to moderate PTB received postoperatively dexamethasone 3 × 8 mg per day. Patients with severe PTB received pre- and postoperative dexamethasone 3 × 8 mg per day. In both cases, the dexamethasone dose was gradually reduced.

### Statistical analysis

R version 4.0.3 and SPSS version 26 software were used for the statistical analyses [27]. The influence variable of primary interest was edema volume, which had a mean of 51.95 cm^3^ in all present edemas. This variable was grouped into three categories: no edema, edema volume < 51.95 cm^3^ and edema volume ≥ 51.95 cm^3^. All tests were two-sided, and a significance level of 0.05 was chosen. For univariate analyses comparing the three edema groups, Fisher’s exact test was applied for categorical variables, whereas the Kruskal–Wallis test was chosen for continuous variables (normality could not be assumed). Dichotomous variables with less than 50 occurrences in one of the categories in all patients were not tested for differences between the three edema groups, because the sample size was too low for a meaningful application of Fisher’s exact test.

The preoperative outcomes of symptomatic meningiomas, cognitive deficits, palsy, cranial nerve disorders, seizure and preoperative KPS score and the postoperative outcomes of total postoperative complications and postoperative KPS score were additionally analyzed by multiple logistic regression. Influence variables for all these outcomes were edema group, age, sex, ASA score, tumor site, tumor volume and preoperative KPS score (except for preoperative KPS score as an outcome) and additionally the two variables tumor resection (Simpson grade) and asymptomatic meningiomas for postoperative outcomes. To achieve sufficient sample sizes per category, the following variables were grouped as follows: ASA score (ASA 1, ASA 2, ASA 3 + 4), tumor site (convexity, flax/parasagittal, tuberculum sellae, other), and tumor resection (Simpson I, Simpson II, Simpson III, Simpson IV + V). For the outcome of palsy, the tumor site tuberculum sellae had to be combined with the category “other” because of the sparse occurrence. Model selection in the multiple models was performed by backward selection and a *p* value threshold of 0.05.

## Results

Six hundred ninety-six patients with primary intracranial meningiomas were enrolled in this study. Three hundred sixty-two (52%) patients did not have PTBE, and 334 (48%) patients had PTBE. The mean PTBE volume was 51.95 cm^3^. A total of 207 (29.74%) patients had a PTBE volume lower than 51.95 cm^3^, and 127 (18.24%) patients had a PTBE volume equal to or larger than 51.95 cm^3^. All patient and tumor characteristics are summarized in Tables 1 and 2.

**Table 1:** Clinical characteristics

	All patients No. (%)	No edema No. (%)	Edema < 51.95 cm^3^ No. (%)	Edema ≥ 51.95 cm^3^ No. (%)	*p* value
No. of patients	696	362 (52)	207 (29.7)	127 (18.2)	
Age (median (IQR))	60 (51–69)	**59 (49–67)**	**61 (52–71)**	**65 (54–74)**	**< 0.001**
Mean	59.5	**57.7**	**60.8**	**62.6**	
Sex					**< 0.001**
Female	526 (75.6)	**291 (80.4)**	**159 (76.8)**	**76 (59.8)**	
Male	170 (24.4)	**71 (19.6)**	**48 (23.2)**	**51 (40.2)**	
No pre-existing disease	183 (26.3)	99 (27.3)	47 (22.7)	37 (29.1)	0.364
NK	2 (0.3)	1 (0.3)	1 (0.5)	–	
Pre-existing disease					
Arterial hypertension	300 (43.1)	151 (41.7)	95 (45.9)	54 (42.5)	0.649
NK	2 (0.3)	2 (0.5)	–	–	
Heart insufficiency/arrhythmia	73 (10.5)	35 (9.7)	23 (11.1)	15 (11.8)	0.740
NK	2 (0.3)	2 (0.5)	–	–	
Heart anomaly	22 (3.2)	7 (1.9)	11 (5.3)	4 (3.1)	NK*
NK	2 (0.3)	2 (0.5)	–	–	
COPD/asthma	47 (6.8)	18 (5)	18 (8.7)	11 (8.7)	NK*
NK	2 (0.3)	2 (0.5)	–	–	
Diabetes mellitus	70 (10.1)	29 (8)	27 (13)	14 (11)	0.144
NK	2 (0.3)	2 (0.5)	–	–	
Renal insufficiency	21 (3)	7 (1.9)	9 (4.3)	5 (3.9)	NK*
NK	2 (0.3)	2 (0.5)	–	–	
Cancers	106 (15.2)	53 (14.6)	34 (16.4)	19 (15)	0.864
NK	2 (0.3)	2 (0.5)	–	–	
Endocrine disorder	115 (16.5)	59 (16.3)	38 (18.4)	18 (14.2)	0.630
NK	2 (0.3)	2 (0.5)	–	–	
Depression	31 (4.5)	18 (5)	9 (4.3)	4 (3.1)	NK*
NK	1 (0.1)	1 (0.3)	–	–	
Other comorbidities	274 (39.4)	143 (39.5)	87 (42)	44 (34.6)	0.402
NK	2 (0.3)	2 (0.5)	–	–	
Neurosurgical interventions	31 (4.5)	14 (3.9)	14 (6.8)	3 (2.4)	NK*
NK	1 (0.1)	1 (0.3)	–	–	
ASA score					**0.043**^**§**^
1	**99 (14.2)**	**58 (16)**	**25 (12.1)**	**16 (12.6)**	
2	**397 (57)**	**218 (60.2)**	**113 (54.6)**	**66 (52)**	
3	**180 (25.8)**	**79 (21.8)**	**59 (28.5)**	**42 (33.1)**	
4	**16 (2.3)**	**6 (1.7)**	**7 (3.4)**	**3 (2.4)**	
5	**2 (0.3)**	**–**	**2 (1)**	**–**	
NK	2 (0.3)	1 (0.3)	1 (0.5)	–	

Variables with significant differences between the three groups are highlighted in bold

*No.* number, *NK* not known, *IQR* interquartile range, *p value p* value for differences between the three groups (without and with edema) (Fisher’s exact test for categorical variables, Kruskal–Wallis test for continuous variables)

*Dichotomous variables with less than 50 occurrences in one of the categories in all patients were not tested for differences between the three groups (without and with edema)

^§^ASA scores > 2 were merged for Fisher’s exact test

**Table 2:** Tumor characteristics

	All patients No. (%)	No edema No. (%)	Edema < 51.95 cm^3^ No. (%)	Edema ≥ 51.95 cm^3^ No. (%)	*p* value
Tumor side					0.285
Right	312 (44.8)	165 (45.6)	89 (43.0)	58 (45.7)	
Left	308 (44.3)	160 (44.2)	99 (47.8)	49 (38.6)	
Both	76 (10.9)	37 (10.2)	19 (9.2)	20 (15.7)	
Tumor site					
Convexity	202 (29)	**82 (22.7)**	**74 (35.7)**	**46 (36.2)**	**< 0.001**
Falx/parasagittal	107 (15.4)	**36 (9.9)**	**50 (24.2)**	**21 (16.5)**	**< 0.001**
Sphenoid wing medial	98 (14.1)	55 (15.2)	23 (11.1)	20 (15.7)	0.318
Sphenoid wing lateral	35 (5)	15 (4.1)	6 (2.9)	14 (11)	NK*
Cavernous sinus	19 (2.7)	17 (4.7)	2 (1)	–	NK*
Tuberculum sellae	50 (7.2)	**44 (12.2)**	**4 (1.9)**	**2 (1.6)**	**< 0.001**
Olfactory groove meningiomas	35 (5)	6 (1.7)	13 (6.3)	16 (12.6)	NK*
Tentorium	41 (5.9)	23 (6.4)	14 (6.8)	4 (3.1)	NK*
Intraventricular	9 (1.3)	3 (0.8)	4 (1.9)	2 (1.6)	NK*
Orbital	16 (2.3)	15 (4.1)	1 (0.5)	–	NK*
Petroclival	37 (5.3)	31 (8.6)	6 (2.9)	–	NK*
Cerebellopontine angle	35 (5)	29 (8)	6 (2.9)	–	NK*
Cerebellum convexity	4 (0.6)	1 (0.3)	2 (1)	1 (0.8)	NK*
Foramen magnum	2 (0.3)	–	2 (1)	–	NK*
Clivus	2 (0.3)	2 (0.5)	–	–	NK*
NK	3 (0.4)	2 (0.5)	–	1 (0.8)	
Tumor volume (median (IQR))	13.95 (4.53–38.35)	**5.26 (2.39–11.70)**	**27.10 (13.75–49.15)**	**43.30 (22.55–75.55)**	**< 0.001**
NK	24 (3.4)	24 (6.6)	–	–	
Tumor characteristics					
Tumor margin					**< 0.001**
Regular	290 (41.7)	**230 (63.5)**	**45 (21.7)**	**15 (11.8)**	
Irregular	390 (56)	**119 (32.9)**	**159 (76.8)**	**112 (88.2)**	
NK	16 (2.3)	13 (3.6)	3 (1.4)	–	
Peritumor rim					**< 0.001**
Present	144 (20.7)	**120 (33.1)**	**20 (9.7)**	**4 (3.1)**	
Absent	511 (73.4)	**216 (59.7)**	**178 (86)**	**117 (92.1)**	
NK	41 (5.9)	26 (7.2)	9 (4.3)	6 (4.7)	
Signal intensity on T2-weighted imaging					**0.003**
Hypointense	52 (7.5)	**31 (8.6)**	**14 (6.8)**	**7 (5.5)**	
Isointense	424 (60.9)	**238 (65.7)**	**117 (56.5)**	**69 (54.3)**	
Hyperintense	135 (19.4)	**49 (13.5)**	**49 (23.7)**	**37 (29.1)**	
Mixed**	41 (5.9)	**19 (5.2)**	**15 (7.2)**	**7 (5.5)**	
NK	44 (6.3)	25 (6.9)	12 (5.8)	7 (5.5)	
Tumor enhancement					**< 0.001**
Homogenous	439 (63.1)	**262 (72.4)**	**118 (57)**	**59 (46.5)**	
Heterogenous	234 (33.6)	**83 (22.9)**	**85 (41.1)**	**66 (52)**	
NK	23 (3.3)	17 (4.7)	4 (1.9)	2 (1.6)	
Tumor blood supply					**< 0.001**
Meningeal	271 (38.9)	**233 (64.4)**	**32 (15.5)**	**6 (4.7)**	
Meningeal and pial	371 (53.3)	**102 (28.2)**	**155 (74.9)**	**114 (89.8)**	
Pial	15 (2.2)	**7 (1.9)**	**6 (2.9)**	**2 (1.6)**	
NK	39 (5.6)	20 (5.5)	14 (6.7)	5 (3.9)	

Variables with significant differences between the three groups are highlighted in bold

*No.* number, *NK* not known, *IQR* interquartile range, *p value p* value for differences between the three groups (without and with edema) (Fisher’s exact test for categorical variables, Kruskal–Wallis test for continuous variables)

*Dichotomous variables with less than 50 occurrences in one of the categories in all patients were not tested for differences between the three groups (without and with edema)

**Tumors with extensive signal differences

### Preoperative clinical condition

Of the 696 patients, 627 (90.1%) were preoperatively symptomatic. Patients with PTBE were significantly more likely to be symptomatic than patients without PTBE, 313 (93.7%) vs. 314 (86.7%), respectively (*p* < 0.001). As the PTBE volume increased, the number of symptomatic patients increased dramatically. Specifically, 125 (98.4%) of 127 patients with severe PTBE showed symptoms compared to 188 (90.8%) of 207 patients with small-to-moderate PTBE (Table 3). In addition, the ASA score was significantly higher in patients with PTBE (*p* = 0.042) (Table 1). Preoperatively, cognitive deficits, palsy and seizures occurred significantly more frequently in patients with PTBE (*p* < 0.001). Aphasia and olfactory dysfunction were more frequently present in patients with PTBE than in patients without PTBE (Table 3). However, due to the small number of patients with aphasia and olfactory dysfunction, the significance could not be tested. Furthermore, patients with severe PTBE more often experienced headache, but the difference was not significant. On the other hand, patients without PTBE had cranial nerve disorder significantly more often (*p* < 0.001) (Table 3).

**Table 3:** Preoperative clinical condition, surgical and histopathological results

	All patients No. (%)	No edema No. (%)	Edema < 51.95 cm^3^ No. (%)	Edema ≥ 51.95 cm^3^ No. (%)	*p* value
Symptomatic meningiomas	627 (90.1%)	**314 (86.7%)**	**188 (90.8%)**	**125 (98.4%)**	**< 0.001**
Preoperative symptoms and neurological deficits					
Headache	200 (28.7)	98 (27.1)	57 (27.5)	45 (35.4)	0.188
Cognitive deficits	91 (13.1)	**15 (4.1)**	**32 (15.5)**	**44 (34.6)**	**< 0.001**
Palsy	60 (8.6)	**15 (4.1)**	**27 (13)**	**18 (14.2)**	**< 0.001**
Sensitivity disorder	43 (6.3)	21 (5.8)	17 (8.2)	5 (3.9)	NK*
Cranial nerve disorder	211 (30.3)	**144 (39.8)**	**50 (24.2)**	**17 (13.4)**	**< 0.001**
Olfactory dysfunction	21 (3)	3 (0.8)	9 (4.3)	9 (7.1)	NK*
Seizure	92 (13.2)	**23 (6.4)**	**41 (19.8)**	**28 (22)**	**< 0.001**
Aphasia	42 (6)	7 (1.9)	20 (9.7)	15 (11.8)	NK*
Gait disorder and dizziness	132 (19)	65 (18)	44 (21.3)	23 (18.1)	0.621
Hormonal disorder	5 (0.7)	1 (0.3)	4 (1.9)	–	NK*
Visible swelling	31 (4.5)	28 (7.7)	2 (1)	1 (0.8)	NK*
Tumor resection (Simpson Grade)					**0.025**^**§**^
1	359 (51.6)	**170 (47)**	**118 (57)**	**71 (55.9)**	
2	191 (27.4)	**98 (27.1)**	**60 (29)**	**33 (26)**	
3	25 (3.6)	**18 (5)**	**5 (2.4)**	**2 (1.6)**	
4	80 (11.5)	**51 (14.1)**	**15 (7.2)**	**14 (11)**	
5	7 (1)	**6 (1.7)**	**1 (0.5)**	**–**	
NK	34 (4.9)	19 (5.2)	8 (3.9)	7 (5.5)	
Histology					
Meningothelial	333 (47.8)	**186 (51.4)**	**83 (40.1)**	**64 (50.4)**	**0.026**
Transitional	125 (18)	60 (16.6)	43 (20.8)	22 (17.3)	0.44
Fibrous	88 (12.6)	**56 (15.5)**	**30 (14.5)**	**2 (1.6)**	**< 0.001**
Psammomatous	14 (2)	9 (2.5)	4 (1.9)	1 (0.8)	NK*
Angiomatous	11 (1.6)	4 (1.1)	2 (1)	5 (3.9)	NK*
Secretory	22 (3.2)	10 (2.8)	6 (2.9)	6 (4.7)	NK*
Microcystic	10 (1.4)	2 (0.6)	3 (1.4)	5 (3.9)	NK*
Choroidal	2 (0.3)	–	1 (0.5)	1 (0.8)	NK*
Atypical	57 (8.2)	**18 (5)**	**22 (10.6)**	**17 (13.4)**	**0.004**
Papillary	1 (0.1)	–	–	1 (0.8)	NK*
Anaplastic	8 (1.1)	2 (0.6)	5 (2.4)	1 (0.8)	NK*
NK	25 (3.6)	15 (4.1)	8 (3.9)	2 (1.6)	
WHO Classification					**< 0.001**^$^
1	621 (89.2)	**338 (93.4)**	**178 (86)**	**105 (82.7)**	
2	59 (8.5)	**18 (5)**	**22 (10.6)**	**19 (15)**	
3	10 (1.4)	**3 (0.8)**	**5 (2.4)**	**2 (1.6)**	
NK	6 (0.9)	3 (0.8)	2 (1)	1 (0.8)	
Ki-67 index (median (IQR))	3.0 (2.0–5.0)	**3.0 (2.0–5.0)**	**5.0 (2.0–5.0)**	**5.0 (3.0–10.0)**	**< 0.001**
(Mean)	5.9	**5.1**	**6.6**	**6.9**	
NK (no. of cases)	197 (28.3)	121 (33.4)	42 (20.3)	34 (26.8)	

Variables with significant differences between the three groups are highlighted in bold

*No.* number, *NK* not known, *IQR* interquartile range, *p value p* value for differences between the three groups (without and with edema) (Fisher’s exact test for categorical variables, Kruskal–Wallis test for continuous variables)

*Dichotomous variables with less than 50 occurrences in one of the categories in all patients were not tested for differences between the three groups (without and with edema)

^§^Simpson grades IV and V were merged for Fisher’s exact test

^$^WHO classes 2 and 3 were merged for Fisher’s exact test

In the multiple logistic regression analysis, age, sex, ASA score, tumor site, tumor volume, and preoperative KPS score were included in the model in addition to the PTBE group to examine their impact on preoperative symptoms: cognitive deficits, seizure, palsy, cranial nerve disorders, as well as on symptomatic meningiomas. PTBE, especially severe PTBE, showed a significant association with cognitive deficits and seizures and a significant inverse association with cranial nerve disorders; however, there was no significance for palsy and symptomatic meningiomas (Table 4).

**Table 4:** Multiple logistic regression analyses for preoperative cognitive deficits, seizure, palsy and cranial nerve disorder

	Odds ratio	95% Confidence interval	*p* value
Cognitive deficits			
No edema	Reference		
Edema < 51.95 cm^3^	3.21	1.65–6.22	< 0.001
Edema ≥ 51.95 cm^3^	9.61	5.01–18.41	< 0.001
ASA Score 3, 4	Reference		
ASA Score 1	0.43	0.18–1.01	0.05
ASA Score 2	0.44	0.26–0.76	0.002
KPS preop	0.97	0.94–0.98	0.001
Without significance: age, sex, tumor sit and tumor volume			
Seizure			
No edema	Reference		
Edema < 51.95 cm^3^	3.08	1.66–5.68	< 0.001
Edema ≥ 51.95 cm^3^	3.82	1.89–7.71	< 0.001
Tumor site: convexity	Reference		
Falx/parasagittal	1.11	0.60–2.05	0.73
Tuberculum sellae	0.13	0.01–1.04	0.05
Other	0.42	0.24–0.74	0.002
Tumor volume in cm^3^	0.98	0.98–0.99	0.01
KPS preop	0.96	0.95–0.98	0.001
Without significance: age, sex and ASA score			
Palsy			
Age	1.03	1.00–1.05	0.005
Tumor site: convexity	Reference		
Falx/parasagittal	0.89	0.40–1.95	0.77
Other	0.45	0.23–0.88	0.01
Tumor volume in cm^3^	1.00	1.00–1.01	0.001
KPS preop	0.94912	0.92822–0.97049	< 0.001
Without significance: edema, sex and ASA score			
Cranial nerve disorder			
No edema	Reference		
Edema < 51.95 cm^3^	0.68	0.43–1.05	0.08
Edema ≥ 51.95 cm^3^	0.27	0.15–0.49	< 0.001
Age	0.97	0.96–0.98	< 0.001
Tumor site: convexity	Reference		
Falx/parasagittal	1.62	0.81–3.24	0.16
Tuberculum sellae	20.48	9.11–46.04	< 0.001
Other	5.30	3.17–8.84	< 0.001
KPS preop	0.96	0.95–0.98	< 0.001
Without significance: sex, ASA score and tumor volume			

Final logistic regression models after backward selection

### Postoperative clinical complications

Two hundred fifty-five (36.6%) patients experienced postoperative surgical and systemic complications. PTBE was significantly associated with a higher rate of postoperative complications (*p* < 0.001). Specifically, the rate of complications increased from 28.2% for patients without PTBE to 41.5% for patients with small to moderate PTBE and to 52.8% for patients with severe PTBE. Among the surgical complications, there were higher incidences of bleeding, CSF fistula, palsy, aphasia, delirium and ischemic infarction in PTBE patients (Table 5); of these, only bleeding was significantly associated (*p* < 0.001). Again, cranial nerve disorders were more frequent in patients without PTBE (*p* = 0.024). Systemic complications, such as pneumonia, occurred more frequently in patients with PTBE (Table 5). Due to the small number of cases with pneumonia, significance testing was not performed.

**Table 5:** Postoperative complications

	All patients No. (%)	No edema No. (%)	Edema < 51.95 cm^3^ No. (%)	Edema ≥ 51.95 cm^3^ No. (%)	*p* value
Total postoperative complications	255 (36.6)	**102 (28.2)**	**86 (41.5)**	**67 (52.8)**	**< 0.001**
Surgical complications					
Bleeding	87 (12.5)	**22 (6.1)**	**37 (17.9)**	**28 (22)**	**< 0.001**
NK	3 (0.4)	3 (0.8)	–	–	
CSF fistula	55 (7.9)	28 (7.7)	11 (5.3)	16 (12.6)	0.067
NK	4 (0.6)	4 (1.1)	–	–	
Meningitis	4 (0.6)	2 (0.6)	1 (0.5)	1 (0.8)	NK*
NK	4 (0.6)	4 (1.1)	–	–	
Palsy	22 (3.2)	6 (1.7)	8 (3.9)	8 (6.3)	NK*
NK	4 (0.6)	4 (1.1)	–	–	
Cranial nerve disorder	68 (9.8)	**46 (12.7)**	**14 (6.8)**	**8 (6.3)**	**0.024**
NK	4 (0.6)	4 (1.1)	–	–	
Aphasia	18 (2.6)	4 (1.1)	7 (3.4)	7 (5.5)	NK*
NK	1 (0.1)	1 (0.3)	–	–	
Seizure	28 (4)	10 (2.8)	13 (6.3)	5 (3.9)	NK*
NK	4 (0.6)	4 (1.1)	–	–	
Delirium	13 (1.9)	2 (0.6)	6 (2.9)	5 (3.9)	NK*
NK	4 (0.6)	4 (1.1)	–	–	
Ischemic infarction	14 (2)	2 (0.6)	7 (3.4)	5 (3.9)	NK*
NK	4 (0.6)	4 (1.1)	–	–	
Systemic complications					
Heart attack	1 (0.1)	1 (0.3)	–	–	NK*
NK	1 (0.1)	1 (0.3)	–	–	
Heart failure	5 (0.7)	2 (0.6)	2 (1)	1 (0.8)	NK*
NK	4 (0.6)	4 (1.1)	–	–	
Vein thrombosis	8 (1.1)	4 (1.1)	3 (1.4)	1 (0.8)	NK*
NK	1 (0.1)	1 (0.3)	–	–	
Pulmonary embolism	4 (0.6)	1 (0.3)	1 (0.5)	2 (1.6)	NK*
NK	1 (0.1)	1 (0.3)	–	–	
Pneumonia	9 (1.3)	1 (0.3)	5 (2.4)	3 (2.4)	NK*
NK	1 (0.1)	1 (0.3)	–	–	
Respiratory insufficiency	6 (0.9)	2 (0.6)	3 (1.4)	1 (0.8)	NK*
NK	1 (0.1)	1 (0.3)	–	–	
Renal insufficiency	2 (0.3)	–	1 (0.5)	1 (0.8)	NK*
NK	1 (0.1)	1 (0.3)	–	–	
Endocrine disorder	4 (0.6)	1 (0.3)	1 (0.5)	2 (1.6)	NK*
NK	4 (0.6)	4 (1.1)	–	–	
Pneumothorax	10 (11.4)	3 (0.8)	5 (2.4)	2 (1.6)	NK*
NK	1 (0.1)	1 (0.3)	–	–	
Hospital length of stay (days, median (IQR))	12.0 (8.0–15.0)	**11.0 (8.0–15.0)**	**13.0 (9.0–16.0)**	**14.0 (10.0–17.0)**	**< 0.001**
Mean	12.7	**11.9**	**13.2**	**14.1**	
NK (no. of cases)	8 (1.1)	6 (1.7)	1 (0.5)	1 (0.8)	
Dismissal					**< 0.001**
Dismissal home	538 (77.3)	**303 (83.7)**	**147 (71)**	**88 (69.3)**	
Dismissal to rehabilitation clinic or other hospital	125 (18)	**42 (11.6)**	**50 (24.2)**	**33 (26)**	
NK (no. of cases)	33 (4.7)	17 (4.7)	10 (4.8)	6 (4.7)	
Follow-up (months, median (IQR))	23.7 (5.0–61.6)	25.5 (5.3–66.5)	21.3 (2.9–47.2)	20.9 (5.1–59.2)	0.151
Mean	40.3	42.8	34.9	41.6	
Follow-up (months/maximum)	202.6	202.6	192	184.7	
NK (no. of cases)	47 (6.8)	17 (4.7)	15 (7.2)	15 (11.8)	

Variables with significant differences between the three groups are highlighted in bold

*No.* number, *NK* not known, *IQR* interquartile range, *p value p* value for differences between the three groups (without and with edema) (Fisher’s exact test for categorical variables, Kruskal–Wallis test for continuous variables)

*Dichotomous variables with less than 50 occurrences in one of the categories in all patients were not tested for differences between the three groups (without and with edema)

In the multiple logistic regression analysis, PTBE group, age, sex, ASA score, tumor site, tumor volume, tumor resection and preoperative KPS score were included to examine their impact on postoperative complications. PTBE, particularly severe PTBE, was highly significantly associated with the incidence of postoperative complications (Table 6).

**Table 6:** Multiple logistic regression analyses for postoperative complications and preoperative and postoperative KPS

	Odds ratio	95% confidence interval	*p* value
For postoperative complications			
No edema	Reference		
Edema < 51.95 cm^3^	1.92	1.31–2.81	< 0.001
Edema ≥ 51.95 cm^3^	2.98	1.91–4.64	< 0.001
Age	1.01	1.00–1.03	0.02
Simpson I	Reference		
Simpson II	1.48	1.01–2.15	0.04
Simpson III	4.21	1.79–9.90	0.001
Simpson IV, V	1.73	1.04–2.86	0.03
Without significance: sex, symptomatic meningiomas, ASA score, KPS preop, tumor site, tumor volume and tumor side			
For pre- and postoperative KPS			
KPS preop			
No edema	Reference		
Edema < 51.95 cm^3^	0.26	0.11–0.59	0.001
Edema ≥ 51.95 cm^3^	0.23	0.09–0.56	0.001
Age	0.97	0.94–0.99	0.03
ASA Score 1	4.07	1.14–14.48	0.02
ASA Score 2	3.86	1.96–7.61	< 0.001
Tumor volume in cm^3^	0.99	0.98–0.99	0.02
Without significance: sex, symptomatic meningiomas, Simpson, tumor site and tumor side			
KPS postop			
No edema	Reference		
Edema < 51.95 cm^3^	0.31	0.14–0.67	0.003
Edema ≥ 51.95 cm^3^	0.88	0.33–2.34	0.80
ASA Score 3, 4	Reference		
ASA Score 1	2.15	0.69–6.64	0.18
ASA Score 2	3.35	1.61–6.94	0.001
KPS preop	1.07	1.05–1.10	< 0.001
Without significance: age, sex, symptomatic meningiomas, Simpson, tumor site, tumor volume and tumor side			

Final logistic regression model after backward selection

### Preoperative and postoperative neurological conditions according to the KPS

The preoperative KPS score was significantly lower in patients with PTBE than in patients without PTBE (*p* < 0.001). The postoperative KPS score was higher in all patients. However, patients with small to moderate and severe PTBE still had a significantly lower KPS score than patients without PTBE (*p* < 0.001) (Additional file 1). The KPS score at the last follow-up revealed further improvement compared to the postoperative KPS score and did not show a significant difference between patients with and without PTBE (*p* = 0.636) (Additional file 1).

The multiple logistic regression analysis revealed a significant association between PTBE, small to moderate PTBE and severe PTBE, and preoperative KPS score. However, postoperative KPS, only with small to moderate PTBE had a significant association when compared to non-PTBE (Table 6).

### Edema index (EI)

The mean edema index for the entire cohort was 3.29 (SD ± 4.63). Patients with small to moderate PTBE showed a mean EI of 2.00 (SD ± 2.23), and patients with severe PTBE showed a mean EI of 5.39 (SD ± 6.43). The multiple logistic regression analysis revealed a significant impact of EI only on postoperative KPS (OR = 1.40, 95% CI [1.04–1.88], *p* = 0.02).

### Outcome

Patients with PTBE had a significantly longer length of hospital stay than patients without PTBE (*p* < 0.001). In addition, patients with PTBE needed significantly more postoperative medical support (*p* < 0.001). Although only 42 (11.6%) patients without PTBE were transferred to a rehabilitation clinic or another hospital, 50 (24.2%) patients with small to moderate PTBE and 33 (26%) patients with severe PTBE were transferred postoperatively to a rehabilitation clinic or hospital for further medical support (Table 5).

Eight patients (1.1%) died in the immediate postoperative period. The mortality rates were 1.9% and 1.6% among patients with small to moderate and severe PTBE, respectively, compared to 0.6% of patients without PTBE. At the 16.8-year follow-up, an additional 12 (1.7%) patients died. Again, the mortality rate was higher in patients with small to moderate PTBE (1.4%) and severe PTBE (3.9%) than in patients without PTBE (1.1%) (Additional file 1).

## Discussion

Intracranial meningiomas are slow-growing tumors and, in most cases, benign [1–3, 25, 26]. Due to their slow growth, intracranial meningiomas can grow to a substantial size before the patient shows clinical signs. In addition to their tumor mass, intracranial meningiomas often develop peritumoral brain edema. The incidence of PTBE among intracranial meningioma patients has been reported to be up to 78% [5, 6, 18, 28]. Many factors influencing PTBE have been discussed over the years, often with controversial results. For example, increasing age was proposed by Gurkanlar et al. and Eksi et al. as a risk factor for PTBE [7, 22]. However, many other authors did not find any correlations between age and PTBE [28–30]. In addition, most authors did not identify sex as an influencing factor for PTBE [29–31]. In our large representative cohort, patients with PTBE tended to be older (*p* < 0.001), and male patients were significantly more likely to have PTBE than female patients (*p* < 0.001). Lee et al. reported a significantly higher rate of males (63.2%) than females (30.3%) with PTBE [8].

Some authors did not see a significant association between tumor site and tumor size and PTBE [10–29]. However, in most reported studies, researchers agree on the associations between tumor site, in particular meningiomas of the convexity, parasagittal region, and fossa anterior, and PTBE [5, 11, 14, 30]. Our results showed a significant association between convexity and falx/parasagittal meningiomas and PTBE (*p* < 0.001), which are in consensus with most authors. In addition, patients with olfactory groove meningiomas often presented with PTBE; however, due to the small number of cases, the significance could not be tested in our cohort. On the other hand, skull base meningiomas, such as tuberculum sellae meningiomas, were significantly less likely to develop PTBE (*p* < 0.001).

Intensive research has been conducted on radiological factors influencing PTBE formation. Irregular tumor margins, absent peritumoral rims, hyperintense signal intensity on T2 sequences, heterogeneous tumor enhancement and pial blood supply were found to be associated with PTBE. In a series of 51 patients, Nakano et al. showed positive associations between irregular tumor margins, absent peritumoral rims and hyperintensities on T2 sequences and PTBE [10]. Tamiya et al. reported an association between PTBE and irregular tumor margins, pial blood supply and missing arachnoid layers between the tumor and brain tissue. However, they did not find any relationships between signal hyperintensity on T2 sequences and heterogeneous tumor enhancement and PTBE [5]. In our study, tumor volume, irregular tumor margins, absent peritumoral rim, signal hyperintensity on T2, heterogeneous tumor enhancement and meningeal and pial blood supply of the tumor were all significantly associated with PTBE. Thus, radiological factors do indeed correlate with PTBE and could be used to assess disease severity.

Regarding histology, tumor types such as atypical, rhabdoid, anaplastic and, in particular, uncommon subtypes such as microcystic, secretory and angiomatous, and a higher Ki-67 index have been suggested to be involved in the development of PTBE [5, 8, 9, 30]. Osawa et al. found PTBE in 11 (69%) of 16 patients with uncommon subtypes of WHO grade 1, in 15 (75%) of 20 patients with WHO grade 2 and in all 3 patients with WHO grade 3 meningiomas [11]. However, some studies have not shown the significant impacts of histological findings on PTBE [4, 10, 29]. Sapkota et al. did not see any evidence of the influence of the Ki-67 index on PTBE [30]. In line with previous observations, we found an association between secretory, microcystic and atypical meningiomas (*p* = 0.003) and PTBE. On the other hand, fibrous meningiomas were significantly more often present in patients without PTBE. In addition, the Ki-67 index was significantly higher in patients with PTBE. Thus, PTBE also depends on tumor type and histopathological characteristics.

Many authors confirm that vascular endothelial growth factor (VEGF) is also involved in the development of PTBE. VEGF expression was found to be significantly increased in patients with PTBE [15, 32, 33].

The pathogenesis of PTBE has been fervently studied over the decades. However, very little is known about the clinical effect of PTBE on patients with intracranial meningiomas, and a systematic examination of this matter is urgently needed. Here, we present a systematic examination of patients with intracranial meningiomas to assess the effect of preoperative PTBE on the presentation of pre- and postoperative symptoms, neurological deficits and complications as well as outcomes, taking into account all other relevant influencing factors, such as preexisting diseases and ASA score. In addition, we accurately determined the volume of PTBE in all patients. In most published studies on this topic, researchers have either assessed or measured PTBE according to the maximum diameter and typically included only a few patients.

Zeng et al. analyzed 112 asymptomatic and 401 symptomatic patients with intracranial meningiomas. In their study, 75.3% of symptomatic patients and only 24.1% of asymptomatic patients had PTBE [34]. In our study, 90.8% of patients with small to moderate PTBE and 98.4% of patients with severe PTBE were symptomatic compared to 86.7% of patients without PTBE (*p* < 0.001 for univariate analysis but not significant in the multivariate analysis). The coherence between seizure and meningiomas has been investigated by some authors. Gupte et al. investigated clinical and genomic factors in 394 patients with meningiomas and showed a positive predictive effect of PTBE on preoperative seizures but not on postoperative seizures [35]. In a series of 222 patients with intracranial meningiomas, Lieu et al. reported a significantly increased risk for pre- and postoperative seizures in patients with PTBE [36]. In our study, preoperative seizures were significantly more frequent in patients with PTBE than in patients without PTBE (*p* < 0.001, for univariate and multivariate analyses). Postoperative seizures also occurred more frequently among patients with PTBE. Although seizure has been described by some authors and is also confirmed by our results, it was not the most common clinical manifestation in patients with PTBE in meningiomas. Preoperative headache (*p* = 0.187) and cognitive deficits (*p* < 0.001, for univariate and multivariate analyses) were the most common clinical signs among our patients with PTBE, particularly among patients with severe PTBE (35.4% and 34.6% vs. 27.1% and 4.1% among patients without PTBE, respectively). Simis et al. did not observe an association between PTBE and headache. However, their series contained only 61 patients, and PTBE volume was not measured [17]. In addition, Loewenstern et al. did not observe a coincidence between headache and PTBE. Although they previously determined the PTBE volume, their investigation was conducted on only 112 patients aged 60 years or older [37]. In a series of 57 patients, Bommakanti et al. reported a higher rate of cognitive deficits in patients with frontal meningiomas and PTBE [38]. They showed a significant improvement in cognitive deficits postoperatively. In a very small sample of 21 patients, van Nieuwenhuizen et al. observed lower postoperative cognitive functioning in patients with large PTBE than in patients without or small PTBE [39].

Preoperative palsy was significantly associated with PTBE in our patients in the univariate analysis (*p* < 0.001) but did not show significance in the multivariate analysis. Markovic et al. and Loewenstern et al. reported an increased risk for palsy and aphasia in their patients with PTBE [18, 37]. In contrast, Simis et al. did not find a correlation between palsy and aphasia and PTBE [17]. In our patients, preoperative aphasia more often presented in patients with PTBE. However, due to the small number of patients with aphasia, the statistical significance could not be assessed.

Furthermore, olfactory dysfunction was more frequent preoperatively in our patients with PTBE. This finding can be explained by the fact that olfactory grow meningiomas frequently show large PTBE [40].

In our study, cranial nerve disorders were significantly more often manifested in patients without PTBE (*p* < 0.001, for univariate and multivariate analyses). These findings are explainable by the fact that skull base meningiomas are more often involved in cranial nerve disorders and rarely cause PTBE, as demonstrated here by us and others [11, 30].

In our study, we did not observe a negative effect of PTBE on the extent of tumor resection, as reported in some studies, but in accordance with the data of Simis et al. [17], Vignes et al. revealed difficulties in surgical resection for patients with PTBE [41].

Postoperatively, bleeding and CSF fistula were by far the most common surgical complications among our patients. Postoperative bleeding was significantly increased in patients with PTBE (*p* < 0.001). The increased risk for postoperative bleeding in patients with PTBE has been reported by some authors [18, 37, 41]. Simis et al. reported a CSF fistula rate of 3.3% in their series [17].

In our study, the risk for postoperative palsy, aphasia and seizure was higher in patients with PTBE, but the relative preoperative risks were even higher. Furthermore, delirium and ischemic infarction were more common in patients with PTBE. However, the number of patients was very small. Markovic et al. demonstrated ischemic infarction in 11.3% of patients with PTBE compared to 8% of patients without PTBE [18].

Among the systemic complications, pulmonary embolism and pneumonia were more common in patients with severe PTBE. Again, the number of patients was very small. Simis et al. found hepatic insufficiency in 1.6% of their patients [17]. Eksi et al. reported a significantly increased risk for postoperative systemic complications in elderly patients with PTBE [22].

Our study showed a significantly lower preoperative KPS score in patients with PTBE than in patients without PTBE (*p* < 0.001, for univariate and multivariate analyses). In addition, the postoperative KPS score was significantly lower in patients with PTBE (univariate analysis: *p* < 0.001, multivariate analysis: *p* = 0.003 and 0.806). All patients had a higher postoperative KPS score. In contrast, the KPS score at the last follow-up was not significantly different between the patients with PTBE and those without PTBE, but was significantly improved in all patients. Schwartz et al. noted a larger PTBE in patients aged 80 years or older with a preoperative KPS score of 40 or lower [42]. They also showed a significant association between larger PTBE and improved outcomes at 3 months. Loewensstern et al. did not see an association between preoperative KPS score and PTBE. However, they reported a significantly lower KPS score in their patients 6 months after surgery and a nonsignificant decrease in KPS score at the last follow-up 5 years after surgery [37].

In our cohort, patients with PTBE stayed in the hospital significantly longer than patients without PTBE (*p* < 0.001) and needed significantly more medical support than patients without PTBE (*p* < 0.001). Markovic et al. reported the same results regarding hospital stay (mean was 14.45 days for patients with PTBE and 12.96 days for patients without PTBE) [18]. Vignes et al. also observed an association between PTBE and longer hospital stay (mean: 21.7 days) [41].

In our study, the postoperative mortality rate was higher in patients with small to moderate and severe PTBE than in patients without PTBE. In addition, the mortality rate in the follow-up period was also higher in patients with PTBE. However, the overall mortality rate was low. Sacko et al. revealed a significantly higher mortality rate in patients over 80 years of age with severe PTBE than in patients with moderate or no PTBE, 33% vs. 4% in the first year and 80% vs. 32% during the follow-up period [43]. In contrast, Vignes et al. did not find a significantly increased mortality rate in their group [41].

One limitation of our study is its retrospective nature. However, with the aim of investigating the clinical effect of PTBE in patients with intracranial meningiomas, we present representative results of a single systematic study with complete preoperative and postoperative data and an accurate determination of PTBE volume for each patient. This is a great strength of our study.

## Conclusions

Preoperative PTBE significantly increased the rate of preoperative symptoms and neurological deficits. Furthermore, the postoperative complication rate and thus the need for medical support were significantly higher in patients with PTBE than in those without PTBE. However, surgery led to a significant improvement in clinical conditions and neurological deficits, particularly over time. Therefore, to prevent postoperative complications, patients with PTBE, especially those with severe PTBE, should receive antiedematous treatment for an extended period before surgery. In patients with severe PTBE, additional antiedematous treatment to dexamethasone, such as mannitol, should be considered.

## Supplementary Information

**Additional file 1: Table S1**Preoperative, postoperative and final (last follow-up) KPS.

## Data Availability

All data relating to this research project are available at the corresponding author and can be viewed at any time if desired.
