# Supplementary material for: Impact of peritumoral brain edema on pre- and postoperative clinical conditions and on long-term outcomes in patients with intracranial meningiomas
Source: Eur J Med Res. 2023 Jan 21;28:40. doi: 10.1186/s40001-022-00962-y (PMC9862965; doi:10.1186/s40001-022-00962-y)
Supplement: Supplementary file 1 — Additional file 1: Table S1Preoperative, postoperative and final (last follow-up) KPS. [file 40001_2022_962_MOESM1_ESM.docx]

**Additional file 1.** Preoperative, postoperative and final (last follow-up) KPS

|  | **All patients**  **No. (%)** | **No edema**  **No. (%)** | **Edema <**  **51.95 cm³**  **No. (%)** | **Edema ≥**  **51.95 cm³**  **No. (%)** | **P value** |
| --- | --- | --- | --- | --- | --- |
|  |  |  |  |  |  |
|  |  |  |  |  |  |
| **KPS preop** (median (IQR))  mean | 80.0 (70.0-90.0)  79.96 (SD±12.07) | **80.0 (80.0-90.0)**  **82.77 (SD±11.03)** | **80.0 (70.0-90.0)**  **78.01 (SD±13.19)** | **80.0 (70.0-80.0)**  **75.12 (SD±10.9)** | **<0.001** |
|  |  |  |  |  |  |
| 10 | - | - | - | - |  |
| 20 | 4 (0.6) | 2 (0.6) | 2 (1) | - |  |
| 30 | 2 (0.3) | - | 1 (0.5) | 1 (0.8) |  |
| 40 | - | - | - | - |  |
| 50 | 12 (1.7) | 2 (0.6) | 7 (3.4) | 3 (2.4) |  |
| 60 | 40 (5.7) | 8 (2.2) | 16 (7.7) | 16 (12.6) |  |
| 70 | 147 (21.1) | 66 (18.2) | 43 (20.8) | 38 (29.9) |  |
| 80 | 249 (35.8) | 122 (33.7) | 78 (37.7) | 49 (38.6) |  |
| 90 | 186 (26.7) | 122 (33.7) | 46 (22.2) | 18 (14.2) |  |
| 100 | 54 (7.8) | 39 (10.8) | 13 (6.3) | 2 (1.6) |  |
| NK | 2 (0.3) | 1 (0.3) | 1 (0.5) | - |  |
|  |  |  |  |  |  |
| **KPS postop** (median (IQR))  mean | 90.0 (80.0-90.0)  83.46 (SD±15.53 | **90.0 (80.0-90.0)**  **86 (SD±12.33)** | **90.0 (72.5-90.0)**  **80.68 (SD±19.03)** | **80.0 (80.0-90.0)**  **80.79 (SD±16.26)** | **<0.001** |
|  |  |  |  |  |  |
| 0 | 8 (1.1) | 2 (0.6) | 4 (1.9) | 2 (1.6) |  |
| 10 | - | - | - | - |  |
| 20 | 5 (0.7) | - | 4 (1.9) | 1 (0.8) |  |
| 30 | 2 (0.3) | - | 1 (0.5) | 1 (0.8) |  |
| 40 | 2 (0.3) | 1 (0.3) | - | 1 (0.8) |  |
| 50 | 7 (1) | 2 (0.6) | 4 (1.9) | 1 (0.8) |  |
| 60 | 26 (3.7) | 7 (1.9) | 16 (7.7) | 3 (2.4) |  |
| 70 | 85 (12.2) | 45 (12.4) | 23 (11.1) | 17 (13.4) |  |
| 80 | 157 (22.6) | 72 (19.9) | 39 (18.8) | 46 (36.2) |  |
| 90 | 292 (42) | 161 (44.5) | 88 (42.5) | 43 (33.9) |  |
| 100 | 109 (15.7) | 70 (19.3) | 27 (13) | 12 (9.4) |  |
| NK | 3 (0.4) | 2 (0.6) | 1 (0.5) | - |  |
|  |  |  |  |  |  |
| **KPS at last follow-up** (median (IQR))  mean | 90.0 (90.0-100.0)  88.19 (SD±15.62 | 90.0 (90.0-90.0)  88.84 (SD±12.93) | 90.0 (90.0-100.0)  88.19 (SD±16.07) | 90.0 (90.0-100.0)  86.18 (SD±21.48) | 0.636 |
|  |  |  |  |  |  |
| 0 | 12 (1.7) | 4 (1.1) | 3 (1.4) | 5 (3.9) |  |
| 10 | - | - | - | - |  |
| 20 | - | - | - | - |  |
| 30 | - | - | - | - |  |
| 40 | 1 (0.1) | 1 (0.3) | - | - |  |
| 50 | 2 (0.3) | - | 1 (0.5) | 1 (0.8) |  |
| 60 | 8 (1.1) | 1 (0.3) | 7 (3.4) | - |  |
| 70 | 26 (3.7) | 14 (3.9) | 9 (4.3) | 3 (2.4) |  |
| 80 | 76 (10.9) | 43 (11.9) | 17 (8.2) | 16 (12.6) |  |
| 90 | 294 (42.2) | 177 (48.9) | 72 (34.8) | 45 (35.4) |  |
| 100 | 167 (24) | 78 (21.5) | 57 (27.5) | 32 (25.2) |  |
| NK | 110 (15.8) | 44 (12.2) | 41 (19.8) | 25 (19.7) |  |

No.= number, NK=not known, IQR=interquartile range, P value=P value for differences between the three groups (without and with edema) (Kruskal–Wallis test). Variables with significant differences between the three groups are highlighted in bold.
